# Housing and Husbandry Factors Affecting Zebrafish (*Danio rerio*) Novel Tank Test Responses: A Global Multi-Laboratory Study

**DOI:** 10.21203/rs.3.rs-4849877/v1

**Published:** 2024-10-16

**Authors:** Courtney Hillman, Barbara D. Fontana, Tamara G. Amstislavskaya, Maria A. Gorbunova, Stefani Altenhofen, Karissa Barthelson, Leonardo M. Bastos, João V. Borba, Carla D. Bonan, Caroline H. Brennan, Amaury Farias-Cea, Austin Cooper, Jamie Corcoran, Eduardo R. Dondossola, Luis M. Martinez-Duran, Matheus Gallas-Lopes, David S. Galstyan, Ella O. Garcia, Ewan Gerken, Robert Hindges, Justin W. Kenney, Maxim A. Kleshchev, Tatiana O. Kolesnikova, Adele Leggieri, Sergey L. Khatsko, Michael Lardelli, Guilherme Lodetti, Giulia Lombardelli, Ana C. Luchiari, Stefani M. Portela, Violeta Medan, Lirane M. Moutinho, Evgeny V. Nekhoroshev, Barbara D. Petersen, Maureen L. Petrunich-Rutherford, Angelo Piato, Maurizio Porfiri, Emily Read, Cássio M. Resmim, Eduardo P. Rico, Denis B. Rosemberg, Murilo S. de Abreu, Catia A. Salazar, Thaliana Stahloher-Buss, Júlia R. Teixeira, Ana M. Valentim, Alexander V. Zhdanov, Patricio Iturriaga-Vásquez, Xian Wang, Ryan Y. Wong, Allan V. Kalueff, Matthew O. Parker

**Affiliations:** a Surrey Sleep Research Centre, Department of Clinical and Experimental Medicine, School of Biosciences, University of Surrey, Guildford, Surrey, GU2 7XH, UK; b Laboratory of Experimental Neuropsychobiology, Department of Biochemistry and Molecular Biology, Natural and Exact Sciences Center, Federal University of Santa Maria, Santa Maria, RS 97105-900, Brazil; c Laboratory of experimental models of neuropsychiatric disorders, Scientific Research Institute of Neurosciences and Medicine, Novosibirsk State University, Novosibirsk, Russia; d Ural Federal University, Yekaterinburg, Russia; e Laboratório de Neuroquímica e Psicofarmacologia, Escola de Ciências da Saúde e da Vida, Pontifícia Universidade Católica do Rio Grande do Sul, Porto Alegre, 90619-900, Porto Alegre, RS, BR; f Alzheimer’s Disease Genetics Laboratory, School of Biological Sciences, Faculty of Sciences, Engineering and Technology, The University of Adelaide, North Terrace Campus, Adelaide, SA, Australia 5005; g Childhood Dementia Research Group, College of Medicine and Public Health, Flinders Health and Medical Research Institute, Flinders University, Bedford Park, SA 5042, Australia; h Laboratório de Psicofarmacologia e Comportamento, Departamento de Farmacologia, Instituto de Ciências Básicas da Saúde, Universidade Federal do Rio Grande do Sul, Ramiro Barcelos 2600, Porto Alegre, RS, 90035-003, Brazil; i School of Biological and Behavioural Sciences, Queen Mary University of London, London, E1 4NS, UK; j Molecular Pharmacology and Medicinal Chemistry Lab, Facultad de Ingeniería y Ciencias, Universidad de la Frontera, Temuco, Chile; k Department of Psychology, University of Nebraska at Omaha, Omaha, Nebraska, 68182 USA; l Translational Psychiatry Laboratory, Graduate Program in Health Sciences, University of Southern Santa Catarina (UNESC), Criciúma, SC, Brazil.; m St. Petersburg State University, St. Petersburg, Russia; n Centre for Developmental Neurobiology & MRC Centre for Neurodevelopmental Disorders, King’s College London, London, England, UK; o Department of Biological Sciences, Wayne State University, Detroit, Michigan, 48202, USA; p Neurobiology Program, Sirius University of Science and Technology, Sochi, Russia; q FishLab, Department of Physiology and Behavior, Federal University of Rio Grande do Norte, Natal, Brazil; r Instituto de Fisiología, Biología Molecular y Neurociencias, Consejo Nacional de Investigaciones Científicas y Tecnológicas. Facultad de Ciencias Exactas y Naturales, Universidad de Buenos Aires, Buenos Aires, Argentina.; s Department of Psychology, Indiana University Northwest, Gary, Indiana, 46408, USA; t Department of Mechanical and Aerospace Engineering, Department of Biomedical Engineering, Center for Urban Science and Progress, New York University, Tandon School of Engineering, New York, 11201, USA; u Laboratory Animal Science, Instituto de Investigação e Inovação em Saúde, Universidade do Porto, (i3S), 4200-135 Porto, Portugal; v Department of Biology, University of Nebraska at Omaha, Omaha, Nebraska, 68182 USA; w Graduate Program in Health Sciences, Federal University of Health Sciences of Porto Alegre, Porto Alegre, Brazil; x Western Caspian University, Baku, Azerbaijan; y The International Zebrafish Neuroscience Research Consortium (ZNRC), Slidell, LA 70458, USA

**Keywords:** Data reproducibility, Zebrafish, Anxiety, Novel tank test, Sex differences, Experimental standardization

## Abstract

The reproducibility crisis in bioscience, characterized by inconsistent study results, impedes our understanding of biological processes and global collaborative studies offer a unique solution. This study is the first global collaboration using the zebrafish (*Danio rerio*) novel tank test, a behavioral assay for anxiety-like responses. We analyzed data from 20 laboratories worldwide, focusing on housing conditions and experimental setups. Our study included 488 adult zebrafish, tested for 5 min, focusing on a variety of variables. Key findings show females exhibit more anxiety-like behavior than males, underscoring sex as a critical variable. Housing conditions, including higher stocking densities and specific feed types, influenced anxiety levels. Optimal conditions (5 fish/L) and nutritionally rich feeds (e.g., rotifers), mitigated anxiety-like behaviors. Environmental stressors, like noise and transportation, significantly impacted behavior. We recommend standardizing protocols to account for sex differences, optimal stocking densities, nutritionally rich feeds, and minimizing stressors to improve zebrafish behavioral study reliability.

The growing data repeatability crisis in bioscience, marked by the frequent failure of studies to produce consistent results upon replication,^1–7^ not only represents a significant reputational challenge, but also limits progress in understanding basic biological processes.^5,8–10^ The causes of this crisis are broad and multifaceted, including selective reporting, publication bias, incomplete data reporting and, critically, variability in experimental methods and laboratory conditions.^10–14^ One potential solution to this problem is global collaboration studies, when multiple laboratories from various regions of the globe collectively examine the same research question using local (potentially diverse) experimental setups and testing protocols. ^5^ By systematically assessing the influence of different between-laboratory parameters on a single outcome measure, we can enhance the robustness and generalizability of findings, helping researchers understand factors that impact study outcomes.

The zebrafish (*Danio rerio*) is a powerful vertebrate model widely used to understand and characterize the biology of a range of neuropsychiatric disorders, including anxiety-related disorders.^15–19^ Anxiety disorders rank as the most prevalent mental health conditions globally, affecting over 300 million individuals.^20^ Exacerbated anxiety is very common as a comorbid condition in individuals diagnosed with other psychiatric disorders, such major depression,^21^ bipolar disorder,^22^ and schizophrenia.^23^

One of the most popular behavioral tests to study anxiety-like responses in adult zebrafish is the novel tank (or novel tank diving) test based on the innate defensive geotaxis of zebrafish. The search terms (“novel tank” OR “tank diving” OR “geotaxis”) AND “zebrafish” in the PubMed database ((PubMed.ncbi.nlm.nih.gov, accessed 2024-05-24) identified 354 records published between 2007 (when the first publication using this protocol appeared (Levin et al., 2007)^24^) and May 2024 - an article on average every 2–3 weeks. These studies range from toxicity assessment^24–29^ to the characterization and understanding of psychiatric disorders^30–36^(Fig. 1). Any reviews, pre-prints and original research not using zebrafish or the novel tank test, were excluded from analyses (*n* = 17), leaving 337 original relevant research articles.

The novel tank test exploits the natural tendency of the fish to swim to the bottom of a novel environment, followed by a gradual habituation and increased exploration over time – typically 5–6 min.^24,31,37^ The standard response of zebrafish typically involves an initial phase where animals spend most of the time in the bottom and can show increased anxiety responses, such as freezing. This behavior is then often followed by a habituation phase where fish gradually increase their activity levels and start to explore the more ‘dangerous’ areas of the tank *(i.e*., the top half/third), which in nature, is a more susceptible predation zone.^24,31,38–40^ There are typically one of two endpoints used – either time spent in the top portion of the tank divided in three zones^24,41–43^ or in the top half,^31,37,44–46^ or number of entries into the top zone.^31,37,45^ Often both metrics – time spent in top as a measure of overall anxiety-like behavior, and number of top entries can be used as a measure of exploratory tendency.^31,39,47^ Although zebrafish swim in a three-dimensional space, both two and three-dimensional approaches are utilized for behavioral scoring of the animals. This may lead to inaccurate or unreproducible reporting of individual and social behavior, undermining data integrity, especially related to zebrafish general locomotion, and a possible overestimate of the number of animals required for the studies.^48^

Despite its wide usage in zebrafish neurobehavioral research, there is little consensus concerning the optimal conditions for this task, for example, the test tank sizes, lighting levels, tank color, among several other factors. In addition, there is no information about how housing/husbandry conditions might affect performance and endpoints. Indeed, pre-test housing can strongly affect behavioral performance. For example, housing fish in the same size tank as the tank in which they are tested reduces the novel tank diving effects.^26^ In addition, tank diving effects were mediated by pre-testing group size, with fish housed individually/two to a tank prior to testing showing a less extreme response to novelty as well as sex ratios in housing tanks significantly impacting the novel tank behavior.^49,50^

However, the lack of reporting and consistency in the testing conditions of the novel tank test raises questions about how far the tank set up can affect the responses of fish in this test, as well as other environmental factors related to housing conditions. Therefore, in the present study, twenty laboratories from across the world performed the same experiment to assess the effects of housing conditions and experimental settings on adult zebrafish (n = 24 [12 male, 12 females]) responses to the 5-min novel tank test.

## Results

### Data distribution and differences across laboratories

**Fig. 2.**
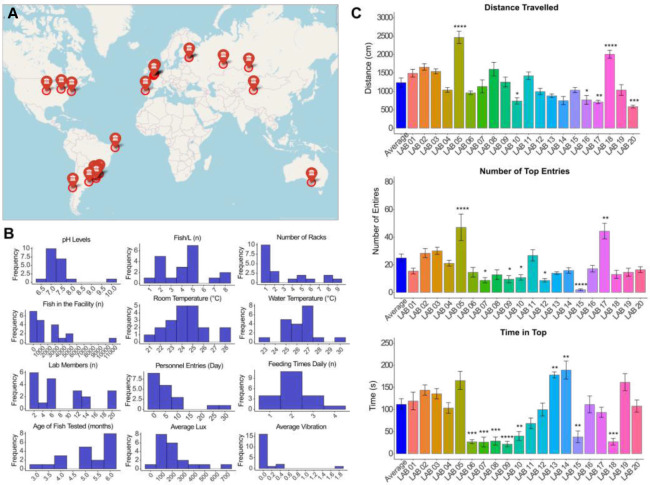
(A) Approximate locations of the different laboratories involved in the data acquisition. An interactive version of this map can be found: https://osf.io/4chwt/?view_only=024aa4208a83420c8fa38e2e0c64943a (B) Distribution of quantitative parameters across laboratories. (C) Differences on main variables comparing each laboratory to the average of the data. Data were represented as mean ± SEM and analyzed by ANOVA followed by Dunnett’s post-hoc test. Asterisks indicate statistical differences compared to the average (*p < 0.05, **p < 0.01, ***p < 0.001 and **** p < 0.0001).

The dataset used in the present study consisted of a total of 2,435 observations and 47 variables, including both categorical and continuous predictors (https://osf.io/4chwt/?view_only=024aa4208a83420c8fa38e2e0c64943a). Initial exploratory data analysis was performed to examine distributions of continuous predictors (Fig. 2B), followed by an ANOVA and Dunnett’s post-hoc comparison test to evaluate significant differences between the laboratories (Fig. 2C). We found that all behavioral parameters (mean distance traveled, time in top and number of entries to the top) varied between the laboratories, with mean distance traveled and number of entries significantly increased for two of the collaborators (p**** < 0.0001; the same collaborators) and decreased for others (p* < 0.05) compared to the average. Number of entries was found higher in two laboratories and lower in another five (p* <0.05). Similarly, two laboratories showed an increase on time spent on top, a parameter usually associated with reduced anxiety-like behavior (p* < 0.05), and the opposite was found for another seven laboratories (p* <0.05). Therefore, this demonstrates the variability of behavioral parameters across the fish tested in these 20 groups. The changes in time spent on top and number of entries across time bins are displayed in **Fig. S1.** We also ran Spearman’s correlations on the average scores per fish (i.e., over the full five-min exposure time) examining the intercorrelation of outcome variables and distance traveled to look for any initial patterns that emerged across the entire sample. There was a weak positive correlation between distance traveled during the test and time spent in the top of the tank (s), (ρ = .14, p** = .002), and a moderate positive correlation between distance traveled during the test and number of top entries, (ρ = .45, p**** < .001). There was also a moderate positive correlation between time in the top (s) and number of top entries, (ρ = .52, p**** < .001). Scatter plots are shown in **Fig. S2.**

**Fig. 3.**
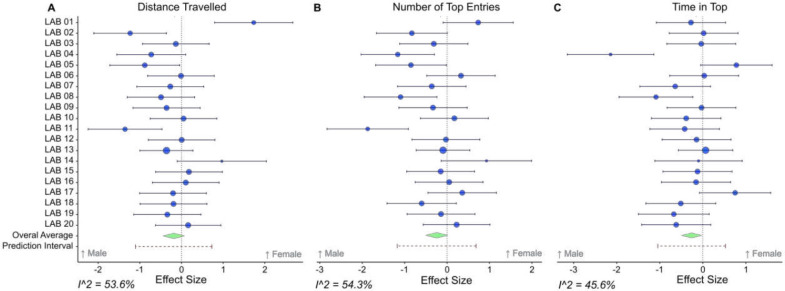
Forest plots illustrating the effect size of A) distance traveled, B) entries to the top zone and C) time in the top zone for various laboratories when comparing females to males. Positive effect size represents higher values for females, and a negative effect size represents higher values for males. Each row represents a different lab, with the overall estimate shown at the bottom calculated using a random effects method. The size of each square is proportional to the weights of each effect size of each laboratory and the arrows indicate the confidence intervals of each lab effect size.

We next considered overall sex differences, as it has already been reported to affect zebrafish anxiety- and activity-related behavioral endpoints (see above). Fig. 3 shows forest plots representing the effect sizes across laboratories, when comparing females to males. We found no significant effect of sex on distance traveled (−0.19, 95% CI [−0.46, 0.08]) (Fig. 3A), entries to the top (−0.25, 95% CI [−0.52, 0.03]) (Fig. 3B) or time spent in the top zone (−0.26, 95% CI [−0.51, −0.01]) (Fig. 3C).

Based on the initial descriptive analysis, the dataset showed moderate heterogeneity (I^2^) values of 53.62, 54.28 and 45.61 for the effect size of distance traveled, entries to the top zone and time spent in the top zone, respectively. To better understand these discrepancies and identify the most influential predictors, we next employed Lasso regression, which helps in selecting and regularizing variables to improve model accuracy and interpretability.

### Analysis of variability in laboratory conditions and experimental settings

To understand the laboratory conditions and experimental settings that can influence the variability in data, Lasso regression was performed, and data is described below. Lasso regression was chosen as it effectively handles high-dimensional data (i.e., datasets with many predictors) and selects relevant predictors by shrinking less important coefficients to zero, thereby enhancing the model’s interpretability and predictive accuracy despite the diverse (and potentially collinear) variables present in the dataset. Mixed-effects models were first fitted to account for the hierarchical structure of the data, with random effects for Lab ID nested within Fish ID. Residuals from these models were extracted for Time in top (s) and top entries to be used as response variables and distance travelled was used as a covariate in subsequent analyses. Design matrices were created for different sets of fixed effects, including main effects, ‘Sex’, ‘Time’ and ‘Sex × Time’ interactions. Lasso regression models were fitted, and cross-validation was used to determine the optimal lambda values to prevent overfitting (**Fig. S3**).

To examine model fit, we calculated the deviance explained by each model and to examine model parsimony we calculated the Akaike Information Criterion (AIC) (Table 1).

Based on the values, the ‘Time’ interaction model was selected for both Time in top (s) and number of Top entries, as it had the lowest AIC and the highest deviance explained for number of Top entries, as well as the lowest AIC and a high deviance explained for the Time in top, indicating the best model for both fit and parsimony. Bootstrapping with 1,000 iterations was then performed for each model to estimate the variability of the coefficients and generate 95% CIs (**Fig. S4**).

For time spent in the top (s), the ‘Time’ interaction model (Deviance Explained = 6.9%, Optimal Lambda = 0.01) revealed several non-zero predictors including sex, number of fish/L in the housing room, total number of racks and whether the fish were fed rotifer diets. There were also several non-zero interactions with time, including the pH, number of fish/L in the housing room, number of racks in the housing room, whether the racks were static or circulating, total number of fish, water temperature, lux levels, vibration, noise levels, wall colour and tank colour, number of lab members, personnel entries, behavior testing in a separate room, feed types, and various sex-time interactions as significant predictors. Similarly, for the number of top entries model (Deviance Explained = 18.33%, Optimal Lambda = 0.01), non-zero predictors in the time interaction model included sex, number of fish/L, whether the racks were circulating, number of fish, vibration, noise levels, tank and floor colour, gender of the personnel, feed types and times, and various time interactions. Finally, significant predictors for both time spent in the top and number of top entries were calculated based on 95%CI not including 0, and these are displayed in **Fig. S5**.

For the time spent in the top of the tank (s), the models revealed several main effects and interactions (**Fig. S4**). In terms of main effects, once we controlled for random effects (and as we saw suggested in the overall models), female fish spent less time in the top section compared to males (**Fig. S4A**). Additionally, the use of rotifer feed was associated with more time spent at the top. A greater number of racks in the facility was associated with less time spent in the top section, whereas a higher density of fish/L was associated with more time spent at the top. Several interaction effects were observed, which identified factors that changed their influence over the 5 minute exposure time (**Fig. S4B**). First, there were two factors that were associated with a negative effect over time (i.e., the variable influence gradually decreased for time spent in top of the tank). For example, while the presence of rotifer feed initially increased the time spent at the top, this effect diminished over time, as indicated by a negative interaction between rotifer feed and time. Similarly, the number of fish in the facility was initially predictive of more time spent in the top, but this effect weakened over time. Other interactions showed a positive effect over time (i.e., had an increasing effect the more time the fish spent in the top of the tank), including pH, and whether they were transported to another room for testing (**Fig. S4B**).

For the number of top entries, the models identified that a higher density of fish/L yielded more top entries, suggesting more exploration. Higher overall noise in the aquarium was associated with fewer top entries (**Fig. S4C**). However, this effect of noise was time-dependent, with the interaction of noise and time in the test showing a positive effect over time (i.e., noise had a stronger effect on top entries as time went on) (**Fig. S4D**).

Finally, the significant predictors identified by the Lasso regression were then used to construct new models to validate their influence on the original Linear Mixed Models (LMM). For the Time in Top parameter, these models included sex, number of racks in the facility, number of fish/L, whether the fish were fed rotifers, the number of personnel entries, the pH of the housing room, and finally, whether behaviour testing was carried out in a separate room. All were run as interactions with Time as an interaction factor (Table 2). Next, for the number of Top entries model, variables chosen were number of fish/L, and noise (dB) in the housing room, both interacting with time (Table 3).

**Fig. 4.**
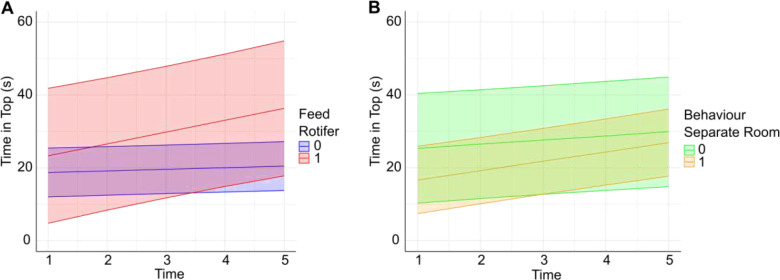
Interaction between rotifer feed (1 = yes, 0 = no) and time (min 1–5 in test) for time in top (s) (A). Interaction between transport prior to testing (1 = yes, 0 = no) and time (min 1–5 in test) for time in top (s) (B). Sixteen laboratories performed the test in a separate room and two laboratories fed rotifers.

Validation of the Lasso regression predictors with the original LMMs revealed several variables that significantly affected both aspects of tank diving performance (time in top and number of entries) (Fig. 4). These included the overall size of the facility, which was associated with less time spent in the top overall, the presence of rotifers in the diet (which was associated with more time in the top, indicative of lower levels of anxiety-like behavior), and whether the fish were transported to the behavior testing room, which was also associated with less time in the top (i.e., increased anxiety-like behavior). In addition, the impact of noise on behavioral outcomes in the novel tank test was confirmed (Table 3). However, both of these latter effects (the rotifers and transport) were only significant in the interaction with time. Fig. 4 characterizes the interaction effects.

## Discussion

The present study sought to examine the factors that underlie variability in adult zebrafish behavior across different laboratories, concentrating on one of the most widely used protocols for measuring anxiety-like behavior, the novel tank test. Our results highlight that the impact of laboratory-specific conditions and experimental settings on experimental outcomes in the novel tank test are relatively minor in nature. These factors generally do not strongly affect the outcome of the study, indicating that fish will tend to dive to the bottom of the novel environment and gradually rise to the top to explore over a 5-min period regardless of experimental factors. As such, time in the test was critical across all laboratories. There was some variability in distance traveled, which varied between laboratories independently and did not correlate with other response variables (Fig. 2). However, we did find that the correlation between distance traveled and number of top entries was considerably stronger than between distance traveled and time spent in the top, suggesting first that these measures should not be used indiscriminately interchangeably, and perhaps that number of top entries is less indicative of anxiety-like behavior than time spent in the top (i.e., it may be more indicative of general movement) (**Fig. S2**).

We used a Lasso regression to identify specific laboratory conditions and experimental settings that significantly influence responses in the novel tank test. We then validated these predictors with LMMs. Several predictors remained significant in the validation models, including type of feed, overall size of the facility, and environmental factors such as noise. Several interaction effects over time were observed, indicating that the factors affected habituation to the test.^49^ In summary, some housing and husbandry conditions, such as specific feed types and not transporting the animals to another room prior to testing, reduced levels of anxiety-like behavior. Background noise was found to be the most significant variable affecting top entries, with higher noise levels leading to more top entries and this effect increasing over time. Noise is usually overlooked in fish studies but was recently shown to increase anxiety-like behavior in zebrafish.^51^ Collectively, this intricate and complex interaction of factors with time confirms the critical importance of including time as a covariate in analyses, even if it is not treated as a main effect.^49^

Although the Lasso models identified sex differences, which have been reported previously and were observed in some laboratories (Fig. 3), this was not seen in the validation models suggesting it is inconsistent. Previous research indicates that female zebrafish tend to show higher levels of anxiety-like behavior than males due to a combination of behavioral, neurobiological, social and pharmacological factors.^52–55^ However, the sex differences are shown to be parameter-dependent with not all reported findings showing sex differences.^56^ Additionally, previous data suggests female zebrafish have increased sensitivity to environmental stressors, in which social and predator stimuli elicit higher anxiety-like responses, also reinforcing the role of environmental cues in influencing anxiety-related phenotypes.^57^ Reasons for these differential anxiety responses may rest in neurobiological differences.^58^ For example, female zebrafish have higher glucose mobilization to hypothalamic brain areas and a distinct pattern of adrenoceptor expression when compared to males, which might contribute to their heightened anxiety-like responses.^59^ There is an extensive literature on the importance of including sex differences in analyses of animal models of neuropsychiatric disorders.^60^ Despite the variability between laboratories in sex effects, the findings further demonstrate the necessity of accounting for sex as a biological variable in behavioral research to ensure the robustness and reproducibility of experimental results.

Several other husbandry factors were associated with differences in novel tank test responses. Although these factors did not have a strong effect on overall tank diving, they did explain some of the variance in these responses. For example, fish held at higher stocking densities spent more time in the top, suggesting lower anxiety-like behavior. Here, the data from the 20 participating laboratories show stock densities ranging from 1 to 8 fish/L, with an average of 3 and 4.75 fish/L for static and recirculating tanks, respectively. While high stocking densities can lead to increased stress among zebrafish, this is only likely to be at the extremes which were not observed in our dataset.^49,61,62^ However, lower densities, while reducing physical crowding, can also lead to stress due to increased visibility of conspecifics, potentially increasing aggression and anxiety-like behavior.^63^ Zebrafish at lower densities may exhibit higher cortisol levels and more aggressive behavior, which can be associated with pronounced anxiety-like responses, such as tank diving.^61,62^ It is worth noting that although there is a lack of reporting on the interaction between rack type (static or recirculating) and stocking density, it is likely that this plays a significant effect on behavioral outcomes. Therefore, it is important to emphasize that very high or very low stocking density can lead to higher anxiety-like behaviors, but as has been previously reported, stocking densities of around 5 fish/L appear to be optimal in terms of the anxiety-like responses in zebrafish. We also report a significant effect of the size of facility on the time in the top with increased facility size resulting in time-dependent heightened anxiety-like responses (reduced time in top) (**Fig. S4B**). Thus, it is apparent that the size of facility and stocking densities play a crucial role in zebrafish novel tank test behavior.

Feed type was an additional predictor, but the data were not clear. We observed that fish that were fed rotifers showed reduced anxiety-like responses. However, it is important to note that (although this was weighted in the statistical models), only two laboratories reported using this feed type with one reporting feed during developmental stages and the other throughout fish life. Therefore, the extent to which rotifers impact larval development and subsequently novel tank test behavior remains a preliminary finding due to many fish being obtained commercially. However, rotifers, as well as other food sources, are a rich source of essential nutrients, including omega-3 and omega-6 fatty acids, proteins, and vitamins, which could support overall health and reduce stress-related behaviors.^64^ In addition, the provision of live feed ensures that zebrafish have access to a continuous and readily available food source, which stimulates the natural environment of fish.^65^ This would likely reduce the stress associated with hunger and food competition, leading to lower levels of anxiety-like behavior.^66,67^ It should also be noted that introducing live food into an animal’s diet could serve as both nutritional and sensory enrichment.^68^ However, interestingly, there was a Time × Rotifer interaction, with the positive effects reducing as a function of time in the test, suggesting that they were initially less stressed, but this ‘normalized’ over time. Thus, the constant presence of food in the maintenance environment could highlight the novelty of the test apparatus by leading to food seeking and consequently increasing anxiety over time. Collectively, these findings suggest that developmental feed type, specifically rotifers as no other commonly used feed type had an impact, may be important in predicting tank diving performance and should be accounted for in studies. This is a hypothesis at this stage, however, because we do not have sufficient evidence to suggest any feeding regimen is preferable. Optimal zebrafish feed is very poorly understood, and the findings here further underscore the importance of working as a community to understand the best feeds for our animals.^69^ This will be a critical area for future work aiming to elucidate how distinct feeding protocols influence anxiety in zebrafish research.

Transporting fish into a different room for behavioral testing was a significant predictor of time spent in the top of the tank. Evidence is clear that zebrafish respond strongly to novel environments. For example, zebrafish housed in a familiar environment exhibit lower baseline anxiety and cortisol levels, suggesting that familiar settings reduce stress and anxiety.^70^ In addition, the presence of familiar cues, such as the same water from their home tank, can mitigate the anxiogenic effects of novel settings. For instance, zebrafish tested in home tank water show less anxiety-like behavior compared to those tested in novel water.^71^ Although there is no direct evidence that this is the case in new rooms (i.e., with novel extra-tank cues), it is possible that, similarly to tank water, novel testing environment also generalizes anxiety responses. In addition, this can be a potential limitation of the present study, as we did not ask participating laboratories to control for habituation time in the novel testing room. Thus, future research can examine this aspect directly. In addition to the novel environment, transport stress elevates cortisol levels in zebrafish, indicating an acute stress response, accompanied by increased glucose levels, which are secondary stress markers.^72^ Again, this can represent a limitation here, as we did not ask laboratories for the timing of the transportation to the testing room. Given the observed effects of testing in a novel room, this could be a promising area for future research.

### Limitations

In our study, although we identified numerous factors impacting behavioral outcomes in the novel tank test, several parameters were not included in our evaluation. These omissions present limitations that should be addressed in future research to enhance the robustness and reproducibility of findings.

Firstly, we asked the laboratories whether the behavior was performed in a different room from the housing facility. However, we did not inquire about habituation to the new environment despite previous research emphasizing the importance of allowing habituation time in a new environment prior to behavioral evaluation.^37,49^ Additionally, Fontana et al. (2021) identified significant effects on novel tank test outcomes related to the number of water changes between different recordings.^73^ Unfortunately, we did not collect this information from the laboratories involved in our research. Most of the data we collected were linked to the environmental conditions of the laboratory. However, some factors potentially affecting behavior during the behavioral recording of the novel tank test were not considered. For example, we did not account for the LUX intensity, noise levels, water column depth, and tank color. It is known that different background colors and light intensities can elicit varied responses in zebrafish anxiety assays.^74^ Deeper tanks have also shown greater reliability, with less data variability and reduced anxiety-related changes in animals when compared to shallower tanks.^75^ The differences in water depth could influence data analysis by affecting the size of the top area of the tank. Furthermore, we did not consider the breeding status of the fish, which can significantly impact behavior. Breeding, especially pair breeding, is a stressful event,^45,49^ and if the novel tank test was conducted on recently bred fish, their behavior might differ from those never bred. Furthermore, water chemistry parameters were not considered. Changes in water salinity and the presence of chemosensory cues from conspecifics significantly impact zebrafish behavior.^76^ Additional limitations include the time of day of behavioral experimentation not being standardized, which can affect behavior. The experimenter’s gender, age, and frequency of working with the fish were not controlled for. For example, in rodents, stress-like behaviors have been identified by depending on experimenter identity^77^, whilst zebrafish models may be more resilient to variation in experimenter identity.^78^ As well as the level of zebrafish behavioral experience in the laboratories, based on the PI’s total years of experience, was not considered. The dimensions of the novel tank used for behavioral assays were not standardized, which can influence the stress response. Some groups measure swim behavior in three-dimensions, others in two-dimensions, impacting metrics like exploratory behavior and distance traveled. The strain of zebrafish was not considered, which can significantly impact behavior. Sex ratios during housing were not reported and therefore the effect of an uneven sex split during housing in the facility was not considered. The impact of feeding status on the day of testing, especially for those using flake foods, was not taken into account.^79^ Variations in LUX intensity, noise and vibration measurements due to different iPhone models were not considered, as the application used for measuring these parameters is dependent on phone camera features. Additionally, proportions of tank size relative to animal length were not analyzed.

In conclusion, while our study focused on husbandry parameters, we acknowledge the importance of other test-related parameters and therefore we suggest a similar investigation to this one should be performed related to test parameters.

### Conclusions and practical implications

The present multi-laboratory study aimed to understand the variability in adult zebrafish behavior across different laboratories, focusing on the novel tank test, a common measure of anxiety-like behavior. Our findings indicate that while laboratory-specific conditions and settings have a minor impact on the outcomes, the time spent in the test is critical across all laboratories. Key predictors of behavior included feed type, facility size, noise, and using a separate room to test the animals. Fish fed with rotifers showed reduced anxiety, likely due to the nutritional content and continuous availability of food, although this finding was from two laboratories and included developmental feeding so can only be considered a preliminary finding. Additionally, the effect diminished over time in the novel tank. Transporting fish to a different room increased anxiety-like behaviors, with the effect growing stronger over time. Noise was shown to increase anxiety-like behaviors, however, this was also time-dependent.

This large-scale global collaborative study further supports the importance of considering husbandry factors in behavioral research, as well as the critical role of housing and husbandry conditions. While laboratory-specific conditions have a relatively minor impact, factors such as sex, stocking density, feed type, and testing environment significantly contribute to anxiety-like behaviors in zebrafish. These findings highlight the need for standardized protocols to optimize zebrafish husbandry and ensure robust, reproducible experimental results. In practical terms, researchers should carefully control and report sex differences at the very least. However, it is likely that efforts to maintain optimal stocking densities around 5 fish/L, provide nutritionally rich and continuous feed sources, and minimize the stress associated with transporting fish to new environments, would also be prudent. Additionally, attention should be given to the personnel interactions within housing facilities. By addressing these factors, researchers can improve the reliability and consistency of behavioral studies in zebrafish, ultimately enhancing the validity of this model for studying neuropsychiatric disorders and other conditions.

## Methods

### Animals, experimental design and ethics

All laboratories carried out the work following local and/or national ethical approvals (see full details of ethical approvals and Questionnaires used in the present study in **Supplementary Materials and OSF, respectively)**.

A total of N = 488 experimentally naive adult zebrafish (240 females and 248 males), ranging from 3 to 7 months post fertilization, were tested in 20 different laboratories for 5 min in the novel tank test. A sample size of *n* = 12 per sex was used following initial effect size and power calculations using G*Power^80^ from three original novel tank test papers^24,26,37^ with a calculated power of 0.95 and required sample size of 4. However, we asked each lab to aim to test 12 male and 12 females to account for the potential (thus-far unknown) variance between laboratories. A variety of zebrafish ‘wild-type’ genotypes were included (including AB, TU, SF, or commercially acquired wild-types). The main behavioral parameters collected for data analysis, including an in-depth analysis of housing conditions and experimental setups, are available on the OSF linked to this paper. No animals were removed from the data analysis.

### Measures

We examined variables that may affect zebrafish novel tank performance, including the test apparatus and tested animals, such as, tank size and animal age, since they both can affect zebrafish novel tank test^26,41^. Moreover, gentle handling and fast transfer to the novel tank was considered, to account for possible effects of human interaction and stress in fish, by asking laboratories to describe their fish handling processes in detail.^81^ We also assessed whether behavioral testing was carried out in a separate room, as this can reduce environmental variability and external disturbances, providing more controlled conditions.^37,49^ Automatization was also considered by asking researchers if behavior was recorded automatically or manually. The videos were analysed per minute for 5 min.

The physical structure of the tank environment was assessed by recording the number of racks in the holding facility, whether the rack was static or circulating, and the total number of fish in the facility. Static racks are likely to provide a different environmental experience compared to circulating racks, which can influence water flow, noise/ vibration and oxygenation, potentially affecting fish movement and comfort.^82^ Temperature of the holding room and temperature of the water in the housing room were included as both ambient and water temperatures directly affect fish physiology and behavior, potentially leading to changes in activity levels and stress responses.^83–85^ The pH level of the housing tank was measured as a numerical predictor because the acidity or alkalinity of the water can significantly affect fish health and behavior.^86,87^ The density of fish per litre of water in the housing tank was also considered, as population density can impact stress levels and social behaviors, thereby affecting diving performance.^61–63,88,89^

Environmental enrichment data was included because it can provide stimulation and reduce stress, potentially impacting zebrafish behavior and well-being.^68,90–92^ Lighting conditions are likely to be significantly important for a range of behavioral differences, and thus light levels were measured from various angles, including lux levels at the top, base, front, and rear of the housing tank (‘Light Meter’ https://apps.apple.com/us/app/lux-light-meter-pro/id1292598866). Light intensity and direction can influence fish visibility and orientation^93^ as well as circadian rhythms and sleep^94^. Vibration levels at the top and bottom of the tank were also recorded, as vibrations can impact fish behavior and stress levels, with different effects depending on where the vibrations occur (‘Vibrometer’ https://apps.apple.com/us/app/vibration-meter-seismograph/id1137580201).^95–97^ Noise levels in decibels (dB; ‘Decibel X’ https://apps.apple.com/us/app/decibel-x-db-sound-level-meter/id448155923) in the housing room were also included in our analyses since noise can be a significant stressor for fish, potentially impacting their behavior and physiology.^90,96,97^ Finally, the colour of the floor, walls in the room and the colour of the housing tanks were measured, as these factors can influence the ambient light environment and the visual comfort of the fish. ^54^

We included several dietary factors, including whether the fish were fed brine shrimp (artemia), flake food, bloodworm, rotifers, or pellet food, as diet can impact fish health and behavior.^69,98^ Finally, the frequency of feeding per day was recorded, as feeding schedules can influence activity levels, stress, and routine behaviors.^79,99^ Finally, we included data on some human factors, including the number of lab members working with the fish, the number of personnel daily entries to the fish holding room per day, the average age of the personnel, and whether the personnel were male or female. These factors might correlate fish experience with humans and handling techniques, influencing fish stress and behavior differently.^81,100^

### Procedure

Through personal connected networks, we invited active laboratories around the world that perform adult zebrafish behavioral studies to take part in the study. An interactive global map highlighting each of the universities involved in this collaborative work (Fig. 3A) was produced using a custom Python code using the Folium library for geographic visualisation. The code as well as a link to the interactive version of the map is open to access (https://osf.io/4chwt/?view_only=024aa4208a83420c8fa38e2e0c64943al).

### Analysis and Statistics

A One-way ANOVA was performed to evaluate the mean effects across laboratories on “*Distance travelled (cm)”,* “*Time spent in the top of the tank (s)”* and “*Number of top entries”* followed by a Dunnett’s post-hoc test comparing each laboratory to the average, to evaluate inter-laboratory difference. Importantly, the average was calculated by generating 24 samples that fell into the values of the 20 laboratories and considered max data deviation for each parameter. Next, to evaluate potential sex effects on three parameters assessed in this work, we analyzed the effect size of female*male differences, and these effect sizes were represented through forest plots. Effect sizes were calculated using the standardized mean difference approach. Specifically, we used Cohen’s d to measure the effect size for each study included in the analysis. The weights were calculated as the inverse of the variances of the effect sizes and confidence intervals were calculated using the effect size SE*1.96 for a 95% confidence level. Following heterogeneity analysis, which resulted in moderate values of heterogeneity (I^2^), forest graphs overall average was generated using a random effects model with DerSimonian and Laird approach. This approach was chosen to account for the variability both within and between studies. Distance travelled and exploratory parameters was chosen for the analysis of sex effect size due to previous studies showing that males travel more than females, and females can show higher anxiety-related responses.^16, 55^

To evaluate variables that can influence novel tank behavior, mixed-effects models were fit to extract residuals for the two primary response variables, “Time spent in the top of the tank (s)” (Time Top) and the “Number of top entries” (Top entries). The model structure included random effects for Lab nested within Fish ID. This allowed us to account for the hierarchical structure of the data, ensuring that variability at the ‘Lab’ and ‘Fish’ levels was appropriately modelled. The residuals from these models were then used as response variables in subsequent analyses.

In high-dimensional datasets such as this (i.e., datasets with a large number of variables relative to the number of observations), the extensive number of predictors can lead to challenges including overfitting, excessive complexity, and critically, difficulties in identifying the most important predictors. High-dimensional data therefore requires specialized statistical methods, such as lasso regression, that can perform variable selection and regularization to build more robust and interpretable models.^101^ First, to explore the fixed effects and their interactions, we created several design matrices based on different fixed effects formulas, including main effects, interactions of main effects with ‘Sex’ and ‘Time’, and finally a combined ‘Sex*Time’ interaction model. Lasso regression models were then fitted using the resulting design matrices.^101^ Cross-validation was performed to determine optimal lambda values in order to control the regularisation strength of the lasso models, to prevent overfitting, and to ensure that the models generalized well to new data.^101^ We next performed bootstrapping with 1000 iterations to estimate the variability of the coefficients generated from each lasso model. Using the bootstrapped results, 95% confidence intervals (95%CI) were calculated for the coefficients of the lasso models. Significant predictors were identified based on the coefficients and their CIs. Any predictors for which CIs did not include zero were considered significant. Finally, deviance explained by each model was calculated to assess model fit, and AIC values were computed for each lasso model to evaluate model parsimony, thus balancing model fit and complexity.^102^ All data analysis were carried out in R Studio version 4.4.1 (https://osf.io/4chwt/?view_only=024aa4208a83420c8fa38e2e0c64943a) using several R libraries.^103^ Data manipulation and preprocessing were conducted with the ‘dplyr’ and ‘readxl’ packages, which facilitated the handling of categorical variables and missing data. To check the distribution and identify outliers in our continuous variables, we used the ‘ggplot2’ library for creating histograms and boxplots.^104^ Mixed-effects models were fitted using the ‘lme4’ package to account for random effects, extracting residuals for further analysis. Specifically, we fitted mixed-effects models to the time in the top (s) and number of top entries variables, incorporating random effects for fish ID nested in Lab. We then applied Lasso regression using the ‘glmnet’ package. A design matrix was created for the predictors, and cross-validation was performed to determine the optimal lambda value for the lasso regression models. Separate lasso regressions were conducted for the residuals of time in the top (s) and number of top entries to identify significant predictors. To ensure the robustness of our findings, we performed bootstrapping using the ‘boot’ package, which provided 95% CI for our coefficient estimates. Functions were defined to fit lasso models on bootstrap samples and extract coefficients, and 1000 bootstrap iterations were performed for both response variables. We extracted significant predictors based on non-zero coefficients. Finally, to validate the models, we re-fitted the original LMMs with the predictors that emerged from the lasso regression models.

## Figures and Tables

**Fig. 1:**
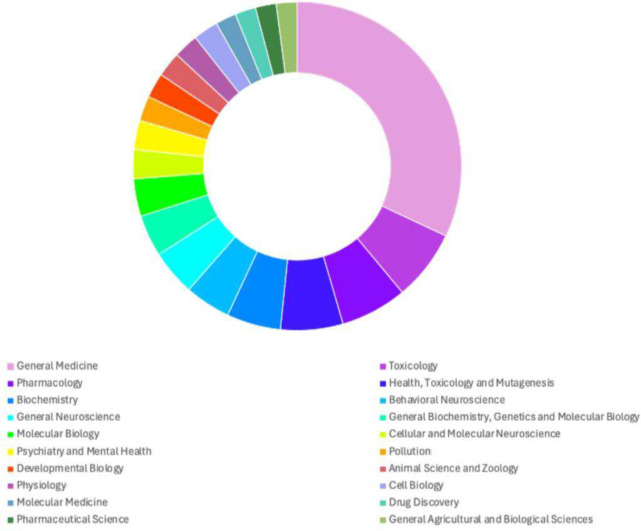
Summary of the different fields of scientific research that have publications using the zebrafish novel tank behavioral assay. This demonstrates the wide and varied use of the assay regardless of the field of study. Data obtained from the PubMed search described previously.

**Table 1:** Deviance Explained and Comparison of AIC for Different Models

Model	Deviance Explained (Time Top)	Deviance Explained (Number top entries)	AIC (Time top)	AIC (Number top entries)

Null	N/A	N/A	18466.26	12885.29
Main	2.83	12.19	18398.98	12607.48
‘Sex’ Interaction	3.13	12.66	18414.79	12618.59
**‘Time’ Interaction**	6.90	18.33*	18371.70*	12444.37*
‘Sex x Time’ Interaction	7.33*	18.04	18441.16	12506.38

**Table 2:** Linear Mixed Models for Time spent in Top (s) with predictors from Lasso Regression.

Predictor	Estimate	Std. Error	df	t value	Pr(>|t|)

(Intercept)	21.72	6.52	14.71	3.33	0.005**
Sex	1.63	0.88	1526.02	1.87	0.062
**Number of racks**	**−2.49**	**1.15**	**14.77**	**−2.16**	0.047*
Fish length	1.79	1.58	14.84	1.13	0.276
Rotifer feed	10.22	8.52	14.67	1.2	0.249
Time	4.46	2.77	1971.6	1.61	0.107
Separate room for performing behavior	−5.83	6.58	14.65	−0.89	0.390
**Time x Rotifer feed**	**3.98**	**1.83**	**1971.66**	**2.17**	0.030*
Time x Number of fish	−0.06	0.25	1971.76	−0.24	0.81022
Time x Personnel entries	0.02	0.55	1971.73	0.05	0.96415
Time x pH	−0.66	0.38	1971.6	−1.76	0.07911
**Time x Separate room**	**2.03**	**0.62**	**1971.84**	**3.28**	**0.00105**

**Table 3:** Linear Mixed Models for Number of Top entries with predictors from Lasso Regression.

Predictor	Estimate	Std. Error	df	t value	Pr(>|t|)

(Intercept)	2.47	1.25	16.72	1.98	0.064
Fish length	0.30	0.27	16.83	1.12	0.279
Noise (dB)	0.31	0.52	16.73	0.60	0.556
**Time**	**0.97**	**0.07**	**1983**	**13.64**	< 2e-16***
**Time x Noise (dB)**	**0.19**	**0.07**	**1983**	**2.67**	0.007**

## Data Availability

All raw data and analysis files for this study are available on the Open Science Framework (OSF). https://osf.io/4chwt/?view_only=024aa4208a83420c8fa38e2e0c64943a

## References

[1] Barch D. M. & Yarkoni T. Introduction to the special issue on reliability and replication in cognitive and affective neuroscience research. Cogn. Affect. Behav. Neurosci. 13, 687–689 (2013). 23922199 10.3758/s13415-013-0201-7

[2] Begley C. G. & Ioannidis J. P. A. Reproducibility in science. Circ. Res. 116, 116–126 (2015). 25552691 10.1161/CIRCRESAHA.114.303819

[3] Branch M. N. The “reproducibility crisis:” Might the methods used frequently in behavior-analysis research help? Perspect. Behav. Sci. 42, 77–89 (2019). 31976422 10.1007/s40614-018-0158-5 PMC6701500

[4] Ioannidis J. P. A. Why most published research findings are false. PLoS Med. 2, e124 (2005). 16060722 10.1371/journal.pmed.0020124 PMC1182327

[5] Korbmacher M. The replication crisis has led to positive structural, procedural, and community changes. Commun. Psychol. 1, 3 (2023). 39242883 10.1038/s44271-023-00003-2 PMC11290608

[6] Open Science Collaboration. Estimating the reproducibility of psychological science. Science 349, aac4716 (2015). 26315443 10.1126/science.aac4716

[7] Ulrich R. & Miller J. Questionable research practices may have little effect on replicability. Elife 9, e58237 (2020). 32930092 10.7554/eLife.58237 PMC7561355

[8] Bernard C. Stop reproducing the reproducibility crisis. eNeuro 10, ENEURO.0032–23.2023 (2023). 10.1523/ENEURO.0032-23.2023 PMC9907389 36746632

[9] Fanelli D. Is science really facing a reproducibility crisis, and do we need it to? Proc. Natl. Acad. Sci. 115, 2628–2631 (2018). 29531051 10.1073/pnas.1708272114 PMC5856498

[10] Samuel S. & König-Ries B. Understanding experiments and research practices for reproducibility: an exploratory study. PeerJ 9, e11140 (2021). 33976964 10.7717/peerj.11140 PMC8067906

[11] Adam D. What reproducibility crisis? New research protocol yields ultra-high replication rate. Nature 623, 467–468 (2023). 37945705 10.1038/d41586-023-03486-5

[12] Baker M. 1,500 scientists lift the lid on reproducibility. Nature 533, 452–454 (2016). 27225100 10.1038/533452a

[13] Francis G. Publication bias and the failure of replication in experimental psychology. Psychon. Bull. Rev. 19, 975–991 (2012). 23055145 10.3758/s13423-012-0322-y

[14] Hunter P. The reproducibility “crisis”. EMBO Rep 18, 1493–1496 (2017). 28794201 10.15252/embr.201744876 PMC5579390

[15] Choi T.-Y., Choi T.-I., Lee Y.-R., Choe S.-K. & Kim C.-H. Zebrafish as an animal model for biomedical research. Exp. Mol. Med. 53, 310–317 (2021). 33649498 10.1038/s12276-021-00571-5 PMC8080808

[16] Fontana B. D. Concomitant taurine exposure counteracts ethanol-induced changes in locomotor and anxiety-like responses in zebrafish. Psychopharmacology 237, 735–743 (2020). 31786647 10.1007/s00213-019-05410-0 PMC7036063

[17] Fontana B. D. Using zebrafish (Danio rerio) models to understand the critical role of social interactions in mental health and wellbeing. Prog. Neurobiol. 208, 101993 (2022). 33440208 10.1016/j.pneurobio.2021.101993

[18] Kalueff A. V., Stewart A. M. & Gerlai R. Zebrafish as an emerging model for studying complex brain disorders. Trends Pharmacol. Sci. 35, 63–75 (2014). 24412421 10.1016/j.tips.2013.12.002 PMC3913794

[19] Mathur P. & Guo S. Use of zebrafish as a model to understand mechanisms of addiction and complex neurobehavioral phenotypes. Neurobiol. Dis. 40, 66–72 (2010). 20493262 10.1016/j.nbd.2010.05.016 PMC3021971

[20] Ferrari A. J. Global incidence, prevalence, years lived with disability (YLDs), disability-adjusted life-years (DALYs), and healthy life expectancy (HALE) for 371 diseases and injuries in 204 countries and territories and 811 subnational locations, 1990–2021: a systematic analysis for the Global Burden of Disease Study 2021. The Lancet 403, 2133–2161 (2024). 10.1016/S0140-6736(24)00757-8 PMC11122111 38642570

[21] Kaufman J. & Charney D. Comorbidity of mood and anxiety disorders. Depress. Anxiety 12, 69–76 (2000). 11098417 10.1002/1520-6394(2000)12:1+<69::AID-DA9>3.0.CO;2-K

[22] Nabavi B., Mitchell A. J. & Nutt D. A lifetime prevalence of comorbidity between bipolar affective disorder and anxiety disorders: A meta-analysis of 52 interview-based studies of psychiatric population. EBioMedicine 2, 1405–1419 (2015). 26629535 10.1016/j.ebiom.2015.09.006 PMC4634892

[23] Buckley P. F., Miller B. J., Lehrer D. S. & Castle D. J. Psychiatric comorbidities and schizophrenia. Schizophr. Bull. 35, 383–402 (2009). 19011234 10.1093/schbul/sbn135 PMC2659306

[24] Levin E. D., Bencan Z. & Cerutti D. T. Anxiolytic effects of nicotine in zebrafish. Physiol. Behav. 90, 54–58 (2007). 17049956 10.1016/j.physbeh.2006.08.026

[25] Bailey J. M. Long-term behavioral impairment following acute embryonic ethanol exposure in zebrafish. Neurotoxicol. Teratol. 48, 1–8 (2015). 25599606 10.1016/j.ntt.2015.01.005 PMC4363207

[26] Bencan Z., Sledge D. & Levin E. D. Buspirone, chlordiazepoxide and diazepam effects in a zebrafish model of anxiety. Pharmacol. Biochem. Behav. 94, 75–80 (2009). 19643124 10.1016/j.pbb.2009.07.009 PMC2771628

[27] Bencan Z. & Levin E. D. The role of α7 and α4β2 nicotinic receptors in the nicotine-induced anxiolytic effect in zebrafish. Physiol. Behav. 95, 408–412 (2008). 18671990 10.1016/j.physbeh.2008.07.009 PMC3174522

[28] Freddo N. Stimulants cocktail: Methylphenidate plus caffeine impairs memory and cognition and alters mitochondrial and oxidative status. Prog. Neuropsychopharmacol. Biol. Psychiatry 106, 110069 (2021). 32800866 10.1016/j.pnpbp.2020.110069

[29] Kulkarni P. Oral dosing in adult zebrafish: Proof-of-concept using pharmacokinetics and pharmacological evaluation of carbamazepine. Pharmacol. Rep. 66, 179–183 (2014). 24905326 10.1016/j.pharep.2013.06.012

[30] De Oliveira J. A comparison of behavioral and reproductive parameters between wild-type, transgenic and mutant zebrafish: Could they all be considered the same “zebrafish” for reglementary assays on endocrine disruption? Comp. Bioch. Physiol. C: Toxicol. Pharmacol. 239, 108879 (2021). 10.1016/j.cbpc.2020.108879 32877737

[31] Egan R. J. Understanding behavioral and physiological phenotypes of stress and anxiety in zebrafish. Behav. Brain Res. 205, 38–44 (2009). 19540270 10.1016/j.bbr.2009.06.022 PMC2922906

[32] Hamilton T. J. Establishing zebrafish as a model to study the anxiolytic effects of scopolamine. Sci. Rep. 7, 15081 (2017). 29118373 10.1038/s41598-017-15374-w PMC5678162

[33] Johnson A. Examining behavioural test sensitivity and locomotor proxies of anxiety-like behaviour in zebrafish. Sci. Rep. 13, 3768 (2023). 36882472 10.1038/s41598-023-29668-9 PMC9992706

[34] Pinheiro-da-Silva J., Agues-Barbosa T. & Luchiari A. C. Embryonic exposure to ethanol increases anxiety-like behavior in fry zebrafish. Alcohol and Alcohol. 55, 581–590 (2020). 10.1093/alcalc/agaa087 32886092

[35] Selvaraj L. K. Baicalein prevents stress-induced anxiety behaviors in zebrafish model. Front. Pharmacol. 13, (2022). 10.3389/fphar.2022.990799 PMC9659741 36386131

[36] Tian D. Enrofloxacin exposure induces anxiety-like behavioral responses in zebrafish by affecting the microbiota-gut-brain axis. Sci. Total Environ. 858, 160094 (2023). 36372168 10.1016/j.scitotenv.2022.160094

[37] Cachat J. Measuring behavioral and endocrine responses to novelty stress in adult zebrafish. Nat. Protoc. 5, 1786–1799 (2010). 21030954 10.1038/nprot.2010.140

[38] Gerlai R., Lahav M., Guo S. & Rosenthal A. Drinks like a fish: zebrafish (Danio rerio) as a behavior genetic model to study alcohol effects. Pharmacol. Biochem. Behav. 67, 773–782 (2000). 11166068 10.1016/s0091-3057(00)00422-6

[39] Haghani S., Karia M., Cheng R.-K. & Mathuru A. S. An automated assay system to study novel tank induced anxiety. Front. Behav. Neurosci. 13, 180 (2019). 31481885 10.3389/fnbeh.2019.00180 PMC6709859

[40] Rosemberg D. B. Behavioral effects of taurine pretreatment in zebrafish acutely exposed to ethanol. Neuropharmacology 63, 613–623 (2012). 22634362 10.1016/j.neuropharm.2012.05.009

[41] Fontana B. D. & Parker M. O. The larval diving response (LDR): Validation of an automated, high-throughput, ecologically relevant measure of anxiety-related behavior in larval zebrafish (Danio rerio). J. Neurosci. Methods 381, 109706 (2022). 36089166 10.1016/j.jneumeth.2022.109706

[42] Fontana B. D., Alnassar N. & Parker M. O. The zebrafish (Danio rerio) anxiety test battery: comparison of behavioral responses in the novel tank diving and light–dark tasks following exposure to anxiogenic and anxiolytic compounds. Psychopharmacology 239, 287–296 (2022). 34651212 10.1007/s00213-021-05990-w PMC8770442

[43] Menegasso A. S. Embryonic exposure to genistein induces anxiolytic and antisocial behavior in zebrafish: persistent effects until the adult stage. Environ. Sci. Pollut. Res. 29, 8957–8969 (2022). 10.1007/s11356-021-16324-w 34498194

[44] Khor B. S., Jamil M. F., Adenan M. I. & Shu-Chien A. C. Mitragynine attenuates withdrawal syndrome in morphine-withdrawn zebrafish. PLoS One 6, 28340 (2011). 10.1371/journal.pone.0028340 PMC3244390 22205946

[45] Onarheim T., Janczak A. M. & Nordgreen J. The effects of social vs. individual housing of zebrafish on whole-body cortisol and behavior in two tests of anxiety. Front. Vet. Sci. 9, 859848 (2022). 35433896 10.3389/fvets.2022.859848 PMC9009241

[46] Tikhonova M. A. A novel laser-based zebrafish model for studying traumatic brain injury and its molecular targets. Pharmaceutics 14, 1751 (2022). 36015377 10.3390/pharmaceutics14081751 PMC9416346

[47] Cachat J. Modeling withdrawal syndrome in zebrafish. Behav. Brain Res. 208, 371–376 (2010). 20006651 10.1016/j.bbr.2009.12.004

[48] Macrì S., Clément R. J. G., Spinello C. & Porfiri M. Comparison between two- and three-dimensional scoring of zebrafish response to psychoactive drugs: Identifying when three-dimensional analysis is needed. PeerJ 7, e7893 (2019). 31637136 10.7717/peerj.7893 PMC6800527

[49] Parker M. O., Millington M. E., Combe F. J. & Brennan C. H. Housing conditions differentially affect physiological and behavioural stress responses of zebrafish, as well as the response to anxiolytics. PLoS One 7, e34992 (2012). 22509375 10.1371/journal.pone.0034992 PMC3324417

[50] Reolon G. K., de Melo G. M., da Rosa J. G. dos S., Barcellos L. J. G. & Bonan C. D. Sex and the housing: Effects on behavior, cortisol levels and weight in zebrafish. Behav. Brain Res. 336, 85–92 (2018). 28822694 10.1016/j.bbr.2017.08.006

[51] Silva P. F., Garcia de Leaniz C., Freire F. A. M., Silveira V. A. M. & Luchiari A. C. Different housing conditions for zebrafish: What are the effects? Behav. Process. 209, 104886 (2023). 10.1016/j.beproc.2023.104886 37150333

[52] Fontana B. D., Cleal M. & Parker M. O. Female adult zebrafish (Danio rerio) show higher levels of anxiety-like behavior than males, but do not differ in learning and memory capacity. Eur. J. Neurosci. 52, 2604–2613 (2020). 31597204 10.1111/ejn.14588

[53] Genario R. Sex differences in adult zebrafish anxiolytic-like responses to diazepam and melatonin. Neurosci. Lett. 714, 134548 (2020). 31629774 10.1016/j.neulet.2019.134548

[54] Marcon M., Benvenutti R., Gallas-Lopes M., Herrmann A. P. & Piato A. What do male and female zebrafish prefer? Directional and colour preference in maze tasks. Eur. J. Neurosci. 56, 4546–4557 (2022). 35831240 10.1111/ejn.15771

[55] Mustafa A., Roman E. & Winberg S. Boldness in male and female zebrafish (Danio rerio) is dependent on strain and test. Front. Behav. Neurosci. 13, 248 (2019). 31803030 10.3389/fnbeh.2019.00248 PMC6877474

[56] Rajput N., Parikh K. & Kenney J. W. Beyond bold versus shy: Zebrafish exploratory behavior falls into several behavioral clusters and is influenced by strain and sex. Biol. Open 11, 8 (2022). 10.1242/bio.059443 PMC9450886 36039864

[57] Amar A. & Ramachandran B. Environmental stressors differentially modulate anxiety-like behaviour in male and female zebrafish. Behav. Brain Res. 450, 114470 (2023). 37148914 10.1016/j.bbr.2023.114470

[58] Wong R. Y., McLeod M. M. & Godwin J. Limited sex-biased neural gene expression patterns across strains in Zebrafish (Danio rerio). BMC Genomics 15, 905 (2014). 25326170 10.1186/1471-2164-15-905 PMC4216363

[59] Ampatzis K. & Dermon C. R. Sexual dimorphisms in swimming behavior, cerebral metabolic activity and adrenoceptors in adult zebrafish (Danio rerio). Behav. Brain Res. 312, 385–393 (2016). 27363927 10.1016/j.bbr.2016.06.047

[60] Kokras N. & Dalla C. Sex differences in animal models of psychiatric disorders. Br. J. Pharmacol. 171, 4595–4619 (2014). 24697577 10.1111/bph.12710 PMC4209934

[61] Andersson M. & Kettunen P. Effects of holding density on the welfare of zebrafish: a systematic review. Zebrafish 18, 297–306 (2021). 34448632 10.1089/zeb.2021.0018

[62] Andersson M. Low holding densities increase stress response and aggression in zebrafish. Biology 11, 725 (2022). 35625453 10.3390/biology11050725 PMC9139139

[63] Sen Sarma O. Optimizing zebrafish rearing – Effects of fish density and environmental enrichment. Front. Behav. Neurosci. 17, 1204021 (2023). 37456810 10.3389/fnbeh.2023.1204021 PMC10340554

[64] Opazo R., Fuenzalida K., Plaza-Parrochia F. & Romero J. Performance of Debaryomyces hansenii as a diet for rotifers for feeding zebrafish larvae. Zebrafish 14, 187–194 (2017). 28192066 10.1089/zeb.2016.1353

[65] Gallas-Lopes M., Benvenutti R., Donzelli N. I. Z. & Marcon M. A systematic review of the impact of environmental enrichment in zebrafish. Lab Anim. 52, 332–343 (2023). 10.1038/s41684-023-01288-w 38017181

[66] Aoyama Y. A novel method for rearing zebrafish by using freshwater rotifers (Brachionus calyciflorus). Zebrafish 12, 288–295 (2015). 25938499 10.1089/zeb.2014.1032 PMC4522954

[67] Lawrence C. The complete and updated ‘rotifer polyculture method’ for rearing first feeding zebrafish. J. Vis. Exp. 107, e53629 (2016). 10.3791/53629 PMC4781655 26863035

[68] Stevens C. H., Reed B. T. & Hawkins P. Enrichment for laboratory zebrafish—A review of the evidence and the challenges. Animals 11, 698 (2021). 33807683 10.3390/ani11030698 PMC8001412

[69] Hillman C., Cooper A., Ram P. & Parker M. O. The effect of laboratory diet and feeding on growth parameters in juvenile zebrafish. bioRxiv (2024). 10.1038/s41684-024-01456-6 PMC11518990 39443748

[70] de Abreu M. S. The impact of housing environment color on zebrafish anxiety-like behavioral and physiological (cortisol) responses. Gen. Comp. Endocrinol. 294, 113499 (2020). 32360541 10.1016/j.ygcen.2020.113499

[71] Tran S., Facciol A. & Gerlai R. Home tank water versus novel water differentially affect alcohol-induced locomotor activity and anxiety related behaviours in zebrafish. Pharmacol. Biochem. Behav. 144, 13–19 (2016). 26921455 10.1016/j.pbb.2016.02.009

[72] Dhanasiri A. K. S., Fernandes J. M. O. & Kiron V. Acclimation of zebrafish to transport stress. Zebrafish 10, 87–98 (2013). 23590401 10.1089/zeb.2012.0843

[73] Fontana B. D., Alnassar N. & Parker M. O. The impact of water changes on stress and subject variation in a zebrafish (Danio rerio) anxiety-related task. J. Neurosci. Methods 363, 109347 (2021). 34478765 10.1016/j.jneumeth.2021.109347

[74] Facciol A., Iqbal M., Eada A., Tran S. & Gerlai R. The light-dark task in zebrafish confuses two distinct factors: Interaction between background shade and illumination level preference. Pharmacol. Biochem. Behav. 179, 9–21 (2019). 30699329 10.1016/j.pbb.2019.01.006

[75] Anwer H. An efficient new assay for measuring zebrafish anxiety: Tall tanks that better characterize between-individual differences. J. Neurosci. Methods 356, 109138 (2021). 33753125 10.1016/j.jneumeth.2021.109138

[76] Mahabir S. & Gerlai R. The importance of holding water: Salinity and chemosensory cues affect zebrafish behavior. Zebrafish 14, 444–458 (2017). 28873052 10.1089/zeb.2017.1472

[77] Katsnelson A. Male researchers stress out rodents. Nature (2014) doi:10.1038/nature.2014.15106.

[78] de Abreu M. S. & Kalueff A. V. Of mice and zebrafish: the impact of the experimenter identity on animal behavior. Lab Anim. 50, 7–7 (2021). 10.1038/s41684-020-00685-9 33299171

[79] Dametto F. S. Feeding regimen modulates zebrafish behavior. PeerJ 6, e5343 (2018). 30090692 10.7717/peerj.5343 PMC6080598

[80] Faul F., Erdfelder E., Lang A.-G. & Buchner A. G*Power 3: A flexible statistical power analysis program for the social, behavioral, and biomedical sciences. Behav. Res. Methods 39, 175–191 (2007). 17695343 10.3758/bf03193146

[81] Tran S. & Gerlai R. The novel tank test: Handling stress and the context specific psychopharmacology of anxiety. Curr. Psychopharmacol. 5, 169–179 (2016).

[82] Lawrence C. & Mason T. Zebrafish housing systems: A review of basic operating principles and considerations for design and functionality. ILAR J. 53, 179–191 (2012). 23382349 10.1093/ilar.53.2.179 PMC4521404

[83] Angiulli E. Increase in environmental temperature affects exploratory behaviour, anxiety and social preference in *Danio rerio*. Sci. Rep. 10, 5385 (2020). 32214187 10.1038/s41598-020-62331-1 PMC7096496

[84] Maffioli E. Brain Proteome and Behavioural Analysis in Wild Type, BDNF+/− and BDNF−/− Adult Zebrafish (Danio rerio) Exposed to Two Different Temperatures. Int. J. Mol. Sci. 23, 5606 (2022). 35628418 10.3390/ijms23105606 PMC9146406

[85] Nonnis S. Acute environmental temperature variation affects brain protein expression, anxiety and explorative behaviour in adult zebrafish. Sci. Rep. 11, 2521 (2021). 33510219 10.1038/s41598-021-81804-5 PMC7843641

[86] Cleal M., Gibbon A., Fontana B. D. & Parker M. O. The importance of pH: How aquarium water is affecting behavioural responses to drug exposure in larval zebrafish. Pharmacol. Biochem. Behav. 199, 173066 (2020). 33137371 10.1016/j.pbb.2020.173066

[87] Zahangir Md. M., Haque F., Mostakim G. M. & Islam M. S. Secondary stress responses of zebrafish to different pH: Evaluation in a seasonal manner. Aquac. Rep. 2, 91–96 (2015).

[88] Gillis D. M. & Kramer D. L. Ideal interference distributions: population density and patch use by zebrafish. Anim. Behav. 35, 1875–1882 (1987).

[89] Shishis S., Tsang B. & Gerlai R. The effect of fish density and tank size on the behavior of adult zebrafish: A systematic analysis. Front. Behav. Neurosci. 16, 934809 (2022). 36275854 10.3389/fnbeh.2022.934809 PMC9581232

[90] Buenhombre J., Daza-Cardona E. A., Sousa P. & Gouveia A. Different influences of anxiety models, environmental enrichment, standard conditions and intraspecies variation (sex, personality and strain) on stress and quality of life in adult and juvenile zebrafish: A systematic review. Neurosci. Biobehav. Rev. 131, 765–791 (2021). 34592257 10.1016/j.neubiorev.2021.09.047

[91] DePasquale C., Fettrow S., Sturgill J. & Braithwaite V. A. The impact of flow and physical enrichment on preferences in zebrafish. Appl. Anim. Behav. Sci. 215, 77–81 (2019).

[92] Gatto E. Environmental enrichment decreases anxiety-like behavior in zebrafish larvae. Dev Psychobiol. 64, 3 (2022). 10.1002/dev.22255 PMC9313885 35312057

[93] Bayarri M. J., Madrid J. A. & Sánchez-Vázquez F. J. Influence of light intensity, spectrum and orientation on sea bass plasma and ocular melatonin. J. Pineal. Res. 32, 34–40 (2002). 11841598 10.1034/j.1600-079x.2002.10806.x

[94] Brüning A., Hölker F., Franke S., Preuer T. & Kloas W. Spotlight on fish: Light pollution affects circadian rhythms of European perch but does not cause stress. Sci. Total Environ. 511, 516–522 (2015). 25577738 10.1016/j.scitotenv.2014.12.094

[95] Febrina R. Modeling the preference of ayu (Plecoglossus altivelis) for underwater sounds to determine the migration path in a river. Ecol. Modell. 299, 102–113 (2015).

[96] Neo Y. Y. Behavioral changes in response to sound exposure and no spatial avoidance of noisy conditions in captive zebrafish. Front. Behav. Neurosci. 9, 28 (2015). 25741256 10.3389/fnbeh.2015.00028 PMC4330796

[97] Wang J. The role of auditory and vibration stimuli in zebrafish neurobehavioral models. Behav. Process. 193, 104505 (2021). 10.1016/j.beproc.2021.104505 34547376

[98] Watts S. A., Powell M. & D’Abramo L. R. Fundamental approaches to the study of zebrafi sh nutrition. ILAR J. 53, 144–160 (2012). 23382346 10.1093/ilar.53.2.144 PMC4064678

[99] Licitra R. Zebrafish feed intake: A systematic review for standardizing feeding management in laboratory conditions. Biology 13, 209 (2024). 38666821 10.3390/biology13040209 PMC11047914

[100] Tran S., Nowicki M., Fulcher N., Chatterjee D. & Gerlai R. Interaction between handling induced stress and anxiolytic effects of ethanol in zebrafish: A behavioral and neurochemical analysis. Behav. Brain Res. 298, 278–285 (2016). 26611561 10.1016/j.bbr.2015.10.061

[101] Ranstam J. & Cook J. A. LASSO regression. Br. J. Surg. 105, 1348–1348 (2018).

[102] Ninomiya Y. & Kawano S. AIC for the Lasso in generalized linear models. Electron J. Stat. 10, 2 (2016).

[103] R Core Team. R: A language and environment for statistical computing. R Foundation for Statistical Computing, Vienna (2021).

[104] Wickham H. Welcome to the Tidyverse. J. Open Source Softw. 4, 1686 (2019).

